# Vibrational Emission Study of the CN and C_2_ in Nylon and ZnO/Nylon Polymer Using Laser-Induced Breakdown Spectroscopy (LIBS)

**DOI:** 10.3390/polym14173686

**Published:** 2022-09-05

**Authors:** Tahani A. Alrebdi, Amir Fayyaz, Amira Ben Gouider Trabelsi, Haroon Asghar, Fatemah H. Alkallas, Ali M. Alshehri

**Affiliations:** 1 Department of Physics, College of Science, Princess Nourah bint Abdulrahman University, P.O. Box 84428, Riyadh 11671, Saudi Arabia; 2 National Centre for Physics, Quaid-i-Azam University Campus, Islamabad 45320, Pakistan; 3 Department of Physics, King Khalid University, P.O. Box 9004, Abha 61413, Saudi Arabia

**Keywords:** LIBS, CN band, polymer, nylon, doped nylon, molecular structure, plasma parameters, laser irradiance

## Abstract

The laser-induced breakdown spectroscopy (LIBS) technique was performed on polymers to study the neutral and ionic emission lines along with the CN violet system (B^2^Σ^+^ to X^2^Σ^+^) and the C_2_ Swan system (d^3^ П_g_–a^3^ П_u_). For the laser-based emission analyses, the plasma was produced by focusing the laser beam of a Q-switched Nd: YAG laser (2*ω*) at an optical wavelength of 532 nm, 5 ns pulse width, and a repetition frequency of 10 Hz. The integration time of the detection system was fixed at 1–10 ms while the target sample was positioned in air ambiance. Two organic polymers were investigated in this work: nylon and nylon doped with ZnO. The molecular optical emission study of nylon and doped nylon polymer sample reveals CN and C_2_ molecular structures present in the polymer. The vibrational emission analysis of CN and C_2_ bands gives information about the molecular structure of polymers and dynamics influencing the excitation structures of the molecules. Besides, it was further investigated that the intensity of the molecular optical emission structure strongly depends on the electron number density (cm^−3^), excitation temperature (eV), and laser irradiance (W/cm^2^). These results suggest that LIBS is a reliable diagnostic technique for the study of polymers regarding their molecular structure, identification, and compositional analysis.

## 1. Introduction

Nylon is a polyamide polymer that is frequently used to make a variety of different types of attire products, such as lithium batteries [1], 3-D printed nylon memory composites [2], asleep bags, rope, seat belts in vehicles, parachuting fabric, tubing pipe, sheets, and dental floss [3]. Unlike other organic or semi-synthetic structures, nylon fibers are completely synthetic. Laser-induced breakdown spectroscopy (LIBS) has been developed as a rapid, reliable, non-destructive, real-time, and in situ detection analytical technique for the spectroscopic study of different organic, inorganic and explosive textiles [4,5,6,7]. LIBS can also be coupled with further spectroscopic techniques, for instance Raman spectroscopy and photoluminescence spectroscopy, to enhance the capacities of LIBS for organic composite analysis [8]. In LIBS, a high-power laser beam is focused through the convex lens on the target surface, which causes laser–matter interaction. This interaction causes high-density micro-plasma containing electrons, ions, and neutral ingredients. The light emitted from this hot plasma shows the spectral identifications of the atomic as well as molecular optical emissions that appear in the sample, which can be used for chemical analysis. For the last couple of years, LIBS has been concerned with polymer composite analyses using spectroscopic information such as CN and C_2_ molecular structures recorded in an open-air environment [9,10]. The molecular optical emission spectra due to the fundamental transition give information about all the polymer composites present in the sample. In atomic emission spectra, a single line transition is produced at a distinct wavelength, whereas in molecular optical emission, a group of spectral lines appear that corresponds to the vibrational modes due to electronic transitions which are distinguished as vibrational emissions of the molecules [11,12]. Although the molecular optical emission band has already been analyzed, however, molecular optical vibrational emission is still to be explored using LIBS. Moreover, the signal-to-noise ratio (SNR) of CN and C_2_ molecular structures is typically the maximum in the spectra of LIBS, revealing suitable evidence for the difference between organic-polymer materials [13].

Formerly reported analyses on the polymers generally focused on molecular emissions and chemometric studies. Numerous research articles have described the study of polymers in different circumstances by applying LIBS-assisted chemometric analysis. Dong et al. [14,15] utilized a laser-ablation molecular isotopic spectrometry (LA-MIS) technique to study the CN and C_2_ molecular optical emission using organic substances. Boueri et al. [16] reported the artificial neural networks (ANNs) coupled with LIBS to distinguish various polymers. Mousavi et al. [17,18] examined the impact of molecular structures and chemical concentrations on C2 and CN spectral emissions in numerous organic substances, including polymers, PAHs, aliphatic carboxylic acids, aromatic carboxylic acids, and amides. Farooq et al. [19] reported the molecular spectral lines of CH, C_2_, CN, and CO molecules along with the LIBS spectra of the elements Ca, Al, Si, Mg, P, Br, and N in polymer samples such as polystyrene and polycarbonate, respectively. Lasheras et al. [20] studied the various polymers using the LIBS technique through normalized coordinates. Costa et al. [21] identified and classified various e-waste polymers by PCA, SIMCA, and KNN based on the LIBS spectra. The proposed SIMCA and KNN techniques can be utilized for the reprocessing of e-waste polymers through different industrial segments. Gregoire et al. [22] investigated the space-time resolved optical spectra of four various kinds of substances, including polyamide with CN bands, polyethylene containing C_2_ bands, and polystyrene comprising two C_2_ bands, in addition to polyoxymethylene, comprising neither CN nor C_2_ bands in laser-produced polymer plasma. Aquino et al. [23] utilized partial least-squares-discriminant analysis (PLS-DA) to detect and distinguish various polymers. More recently, Zhu et al. [24] studied the C_2_ and CN spectral emissions characteristics from various coals and their pyrolysis char target samples. Unfortunately, the above-mentioned literature review shows that the majority of the work was presented to the classification analysis of various polymers. Hence, a qualitative analysis including optical emission spectra and plasma diagnostics as a function of the laser power density of the synthetic polymers plays a major role in characterizing the organic polymers. Taking into account the above-mentioned characterization, the present study establishes the potential of the LIBS technique to perform qualitative analyses together with the plasma characterization of the pure and ZnO/nylon polymer samples.

ZnO has various incomparable properties such as catalytic, optical, and electronic, due to direct-wide bandgap semiconductor having band gap energy (E_g_) ~3.4 eV along with an enormous excitonic binding energy of ~60 meV, signifying that it is an efficient component for luminescent and lasing devices with steady room temperature [25]. The bandgap of ZnO semiconductors is analogous to the bandgap of TiO_2_. ZnO ingredients are reported for the photo degradation of volatile organic compounds, antibiotics, pesticides, dyes, and surfactants. The different polymers doped with ZnO have radiative recombination processes that may be developed for broadband light and wavelength-tunable emission [25] as these processes are affected by various conditions such as the organic and inorganic properties of the elements along with their comparative chemical composition. Nylon 6/6 is useful as a potential substantial in several areas for diverse applications such as in lithium-ion batteries, antibacterial agents, and cement to enhance the properties [26,27,28]. Nylon 6/6 has extraordinary thermal stability, hydrophilicity and excessive tensile power [29]. Nylon 6/6 doped with ZnO (ZnO/nylon) can be employed as a photocatalyst for the photo degradation of alizarin red dye [30].

In the present work, the LIBS spectra of organic materials such as pure nylon and nylon doped with ZnO (ZnO/nylon) have been studied including the vibrational emission of the C_2_ and CN molecular optical bands. The obtained molecular optical emission is established on the optimized experimental parameters such as the integration time, laser pulse energy, and laser pulse irradiance. Laser irradiance performs a significant role in the vibrational transitions observed in the molecular optical emissions. Furthermore, the intensity variation of vibrational transitions and trend of plasma parameters has been investigated by varying laser irradiance for pure and ZnO/nylon polymers. The analysis of vibrational emission of CN (hetero-nuclear diatomic) and C_2_ (homo-nuclear diatomic) molecules in polymers has revealed that LIBS is a fast and consistent diagnostic technique for the study of polymers regarding their molecular structure.

## 2. Materials and Methods

**Figure 1:**
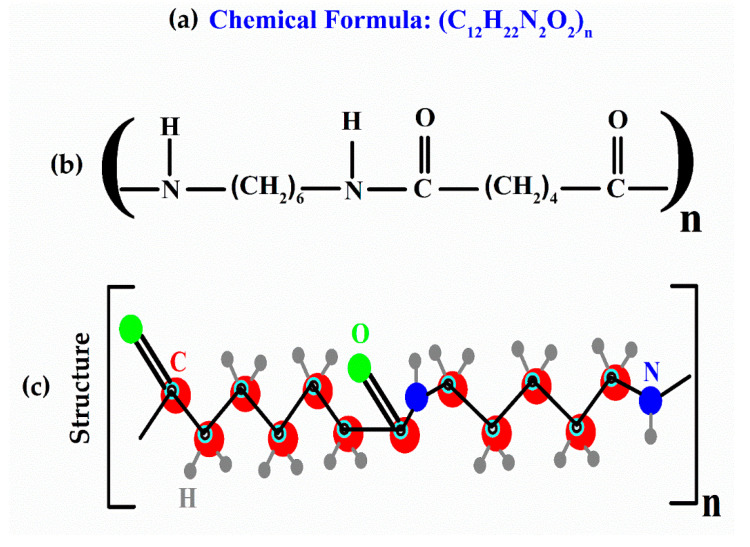
Investigated nylon; (**a**) chemical formula, (**b**) 2D and (**c**) 3D molecular structure.

Two organic polymers were utilized in this study, namely: nylon, and ZnO/nylon. The molecular formula along with the 2D and 3D molecular structure of the nylon organic polymer is presented in Figure 1. Nylon (6/6) and ZnO was obtained from Sigma-Aldrich (USA). The composite ZnO/Nylon polymer film was prepared using solution method technique. 

### Experimental Details

The details of the experimental system utilized to accomplish the optical emission of any sample have been described somewhere else [31,32,33]. In brief, a second harmonic (2ω), Q-switched Nd:YAG laser (Quantel (Brilliant-B), France), was used; it had a 532 nm wavelength and operated at a repetition frequency of 10 Hz with a 5 ns pulse width, and was capable of delivering the pulse energy of about 200 mJ. The LIBS experiments were performed at room temperature in an airy ambiance. The laser beam was converged onto the sample surface, which was positioned in the air at atmospheric pressure using a convex (quartz) lens with a focal length of 10 cm. The samples were kept on a rotating circular stage with a rotation speed of ~10 rpm to enable a clean surface for each fresh laser shot. The optical emission spectra were recorded using an optical fiber attached to a charged coupled devices (CCDs) array spectrometer with an optical wavelength range from 200 nm to 720 nm. An average of ten laser shots were employed to cleanse the surface of the target sample. The spectra were accomplished at an average of 20 laser pulse shots at different spots on the target surface. The averaged optical emission spectrum was then utilized to acquire the molecular optical emission of the polymer samples, which takes into account the target sample inhomogeneity and lowers the statistical inaccuracies.

## 3. Results and Discussion

### Emission Studies of the CN Violet and C_2_ Swan System

**Figure 2:**
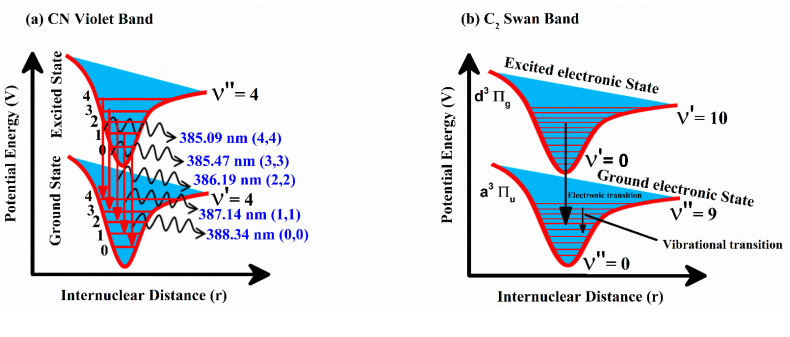
(**a**) Schematic diagram for the spectra of nylon in the CN spectral region (**b**) Potential energy against internuclear distance curves for the two electronic states of the C_2_ Swan band system, revealing the various transitions.

LIBS has been a widely utilized analytical tool for the analysis of synthetic–organic materials such as teflon, epoxy, polystyrene, and nylon. Overall, all organic materials show very analogous molecular or atomic emission spectra. However, various features such as molecular bonding dissociation and vibrational emission can be studied by varying laser irradiance, integration time, and laser power density. Figure 2a shows a schematic transition diagram for the spectra of nylon in the CN spectral region from 385 nm to 388 nm along with (0,0), (1,1), (2,2), (3,3), and (4,4) vibrational transitions at 3.2 eV excitation energy. The molecular optical emission of the CN violet system of a diatomic molecule relates to radiative transitions from B^2^Σ^+^ to X^2^Σ^+^ of the electronic state for ∆ν = 0 at 2.4 eV excitation energy. The transitions from the lowest vibrational states correspond to high probability and strong relative intensity as compared to the highest vibrational states. Figure 2b shows potential energy curves against internuclear distance for the two electronic states of the C_2_ Swan band system also known as vibronic (400–700 nm), revealing the transitions between d^3^ П_g_ and a^3^ П_u_ (from the Plank equation; ΔE = hc/λ). Emitted wavelength λ of the C_2_ molecular optical emission lies between the spectral region from 400 nm to 700 nm. C_2_ Swan system originated due to a variation in wavefunction-symmetry as of the allowed electronic dipole transition between two ^3^П states from upper to lower state (g → u). The Swan band spectrum of C_2_ corresponds Δν = +1, Δν = +2, Δν = 0, and Δν = −1 between excited electronic states to the ground electronic states with ν′= 0–10, and ν″ = 0–9 vibrational transitions. Where, ν″ and ν′ are the vibrational states’ quantum parameters [34] for the lower and upper vibrational stages of the electronic levels, respectively.

**Figure 3:**
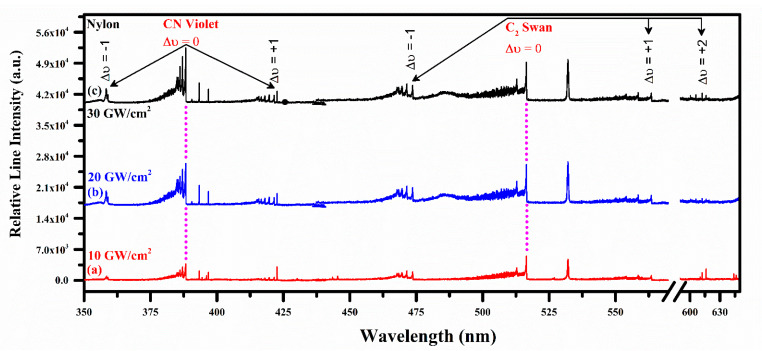
Time-integrated spectrum of the pure organic nylon from 350 nm to 645 nm at different laser irradiance (GW/cm^2^) showing CN and C_2_ vibrational optical emissions.

Figure 3 shows time-resolved LIBS emission spectra of pure nylon in the wavelength spectral region from 350 nm to 645 nm. Nylon spectrum clearly demonstrates the presence of molecular optical emission band of CN Violet at 388.34 (0,0), 387.14 (1,1), 386.19 (2,2), 385.47 (3,3), and 385.09 (4,4) nm due to B^2^Σ^+^–X^2^Σ^+^ and C_2_ Swan band structure at 467.3 (5,4), 468.3 (4,3), 469.8 (3,2), 471.6 (2,1), 473.7 (1,0), 509.6 (2,2), 512.9 (1,1), 516.5 (0,0), 550.2 (3,4), 554.1 (2,3), 558.5 (1,2), 563.5 (0,1), 600.5 (3,5), 606.0 (2,4), and 612.0 (1,3) nm due to d^3^ П_g_–a^3^ П_u_ transitions, respectively. Transitions band heads between two electronic states are identified using the difference in vibrational quantum numbers from the upper to lower electronic state such as Δν = ν′ – ν″. Pure nylon spectra are observed at different laser irradiances such as 10, 20, and 30 GW/cm^2^ and 1–10 ms integration time. Time integrated spectra show continuous growth of the C_2_ and CN molecular bands emission intensities. The strongest transitions of the CN and C_2_ molecular optical emission bands are observed at 388.34 nm (Δν = 0), and 516.5 nm (Δν = 0) wavelength, respectively. Interestingly, the sequence Δν = 0 for the C_2_ structure around 600 nm is shown prominently only at 30 GW/cm^2^ irradiance, showing emission dependency at laser energy accumulation on the target material.

**Figure 4:**
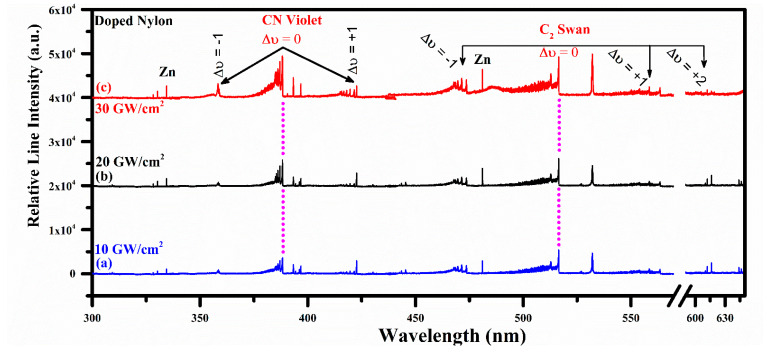
Time-integrated spectrum of the organic doped nylon from 300 nm to 645 nm showing CN and C_2_ vibrational optical emissions together with well-isolated zinc optical spectral lines.

For the comparative molecular optical emission analyses, organic nylon doped with ZnO was also utilized in this study. Figure 4 validates the similar molecular optical emission structures of the CN and C_2_ band at 10, 20, and 30 GW/cm^2^ laser irradiance. However, in this optical region from 300 nm to 645 nm, the expected emission spectral lines of zinc are also observed with an excellent signal-to-noise ratio (SNR) at 328.2, 330.3, 334.5, and 481.1 nm due to 4d ^3^D_1_ → 4p ^3^P_0_, 4d ^3^D_1_ → 4p ^3^P_1_, 4d ^3^D_3_ → 4p ^3^P_2_, and 5s ^3^S_1_ → 4p ^3^P_2_ radiative transitions, respectively. Nevertheless, it is worthwhile to mention that various structures of Ca emission lines at 393.37, 396.85, 442.54, 443.50, 445.48, 527.03, 558.20, 558.88, 559.01, 559.45, 559.85, 612.22, 616.13, and 616.22 nm are identified in the synthetic nylon. Carbon (C) and hydrogen (H_α_) emissions at 247.86 nm (2p3s ^1^P_1_ → 2p^2 1^S_0_) and 656.28 nm (3d ^2^D_5/2_ → 2p ^2^P_3/2_) are also observed. Therefore, carbon (C) is the major source of molecular band formation in nylon. 

## 4. Plasma Characterization

To describe the laser-induced plasma established on the optical emission spectra, specific conditions have to be fulfilled, i.e., the plasma must be optically thin and should follow the local thermodynamical equilibrium (LTE) order. As optically thick plasma would entail self-absorption and saturation in the emitted line profiles causes an irregular peak in the spectrum, it influences the computations for electron plasma density and excitation temperature. We have utilized the line intensity proportion procedure to confirm the condition of an optically thin laser plasma [35,36]:

(1) $$\frac{I_{ki}}{I_{nm}}=\left(\frac{\lambda_{nm}}{\lambda_{ki}}\right)\left(\frac{A_{ki}}{A_{nm}}\right)\left(\frac{g_{k}}{g_{n}}\right)exp\;\left(-\frac{E_{k}-E_{n}}{k_{B}T}\right)$$

where *I_ki_* and *I_nm_* are the detected spectral line intensities at optical wavelength *λ_nm_* and *λ_ki_*, respectively. *A_ki_* and *A_nm_* are the transition probabilities, $g_{k}$ and $g_{n}$ are the statistical weights of the upper levels, *k_B_* is the Boltzmann constant and T is the excitation temperature. We have employed the optical emission lines with nearly the equivalent or very adjacent energies to the upper levels to minimize the temperature influence. The experimentally observed ratio of the spectral line intensities and the ratio determined from the spectroscopic factors are in excellent agreement within a 5% error. For illustration, the experimentally calculated intensity ratio using Zn I at transitions 328.23 nm and 330.29 nm is 0.45, whereas the intensity ratio calculated from the spectroscopic parameters is 0.47. The error relative to the theoretical ratio is found within 5%, which is acceptable. Thus, the optical plasma can be assumed as optically thin.

### 4.1. Plasma Excitation Temperature

**Figure 5:**
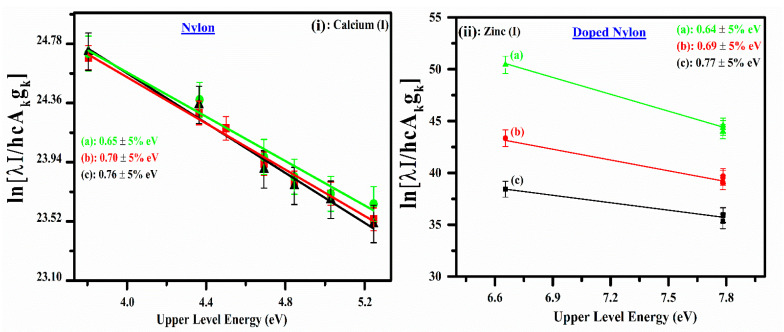
(**i**) Temperature Boltzmann plots for the pure organic nylon drawn using five neutral calcium (Ca I) emission lines. (**ii**) Boltzmann plots for the co-doped nylon made using zinc optically thin emission lines.

The excitation temperature was determined by applying the Boltzmann plot technique, having established that the optical plasma is optically thin. The spectroscopic atomic factors of the optically thin spectral lines of Ca I and Zn I applied to construct the Boltzmann plots were acquired from the NIST database [37]. The spectroscopic parameters of the persistent lines including wavelength (nm), upper-level energy (eV), electron configuration transition, statistical weight, and transition probability (s^−1^) are presented in Table 1. To draw the Boltzmann plots, these lines were carefully chosen as they lie in a thin optical region where the efficiency of the detector device stays nearly persistent, thus reducing the inaccuracies associated with the measurements of the line intensity. The excitation temperatures have been determined using the Boltzmann plot procedure in which numerous spectral emission lines along with their relative line intensities are considered [38,39]:

(2) $$\ln\left(\frac{I_{ki}\lambda_{ki}}{A_{ki}g_{k}}\right)=-\frac{E_{k}}{k_{B}T_{e}}+\ln\left(\frac{FN}{U\left(T\right)}\right)$$

where, $F=\frac{hcd}{4\pi }$, *d* is the characteristic plasma length, *I_ki_* is the spectral line intensity due to the *k → i* transition, $\lambda_{ki}$ is the spectral wavelength, h is the Planks constant, c is the speed of light, $A_{ki}$ is the transition probability, $g_{k}\;$ is the upper-level statistical weight, $E_{k}$ is the upper-level transition energy. Similarly, $k_{B}$, *T_e_*, *N*, and U(T) are the Boltzmann constant, excitation temperature, total number density, and the temperature-dependent partition function, respectively. A plot corresponding to the logarithmic term in this equation versus the upper-level energies (*E_k_*) yields a straight line (y = mx +c) and its slope (m) is like $1/kT_{e}$. The Boltzmann plots corresponding to the Ca I, and Zn I lines for pure nylon and doped nylon, respectively, are shown in Figure 5, demonstrating good linearity; the linear correlation coefficients are: $R_{Ca}^{2}≻0.95$, and $R_{Zn}^{2}≻0.97$, respectively. The excitation temperatures have been determined from the slopes of the lines corresponding to Ca I, and Zn I. The excitation temperature at different irradiances (10–30 GW/cm^2^) was calculated as; 0.65 eV to 0.76 eV for pure nylon and 0.64 eV to 0.77 eV for doped nylon. The error bars show the uncertainty (~5%) in the measurement of electron temperature, which is attributed to the error in the integrated line intensity, transition probability, and the line profile fitting method. 

### 4.2. Electron Number Density

**Figure 6:**
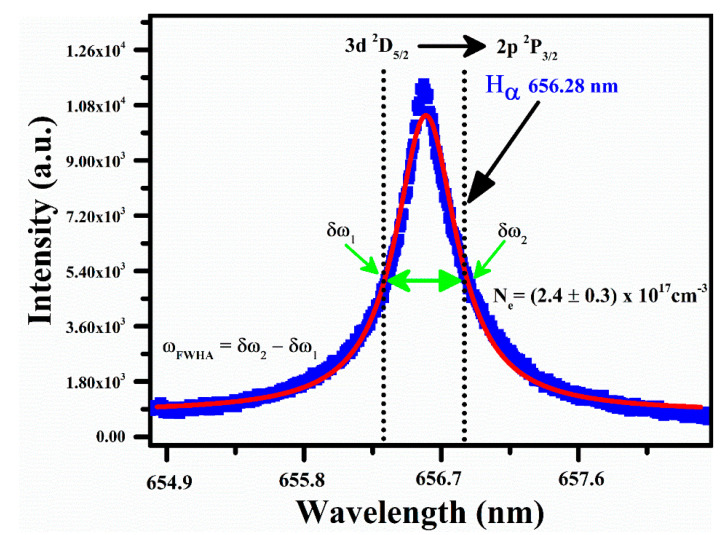
Stark broadened line profile of hydrogen H_α_ spectral line profile at 656.28 nm through the Voigt fitting.

The full width at a half area (*FWHA*) is calculated using a hydrogen H_α_ spectral emission line profile at 656.28 nm as demonstrated in Figure 6, displaying the experimentally analyzed line profile (blue color) corresponding to *FWHA* yielding as (0.99 ± 0.05) nm. The simplest formula for the scheming of electron number density using the H_α_ line is given as [40,41]:

(3) $$\omega_{FWHA}=5.49\;Å\times {\left(\frac{N_{e}}{10^{17}{\mathrm{cm}}^{-3}}\right)}^{0.67965}$$

Here, *N_e_* is the plasma number density, and ω_FWHM_ is the full width at the half area. The parameter *ω_FWHM_* is calculated through the relation, $\omega_{FWHA}=\delta \omega_{2}-\delta \omega_{1}$. The calculated electron number density using Equation (3) is $\left(2.4\;\pm \;0.3\right)\times 10^{17}$ cm^−3^. The error bars show the uncertainty (∼5%) in electron density, which is principally owing to the error in the electron impact parameter, *FWHM*, and in the deconvolution of the line width to the instrumental width.

### 4.3. Local Thermodynamical Equilibrium (LTE) Condition

The ionic and the excitation temperatures have come to be identical to the electron temperature for the optical plasma to be in local thermodynamical equilibrium (LTE). McWhirter advised a basis for a trivial limit of the electron number density to confirm the plasma to be in LTE. The above criteria was validated by calculating the smaller limit of the electron plasma density using the following relation [42,43]:

(4) $$N_{e}\left(cm^{-3}\right)\geq \;1.6\times 10^{12}\sqrt{(T_{\left(K\right)}}{\left(\Delta E_{nm}\;\left(eV\right)\right)}^{3}$$

where, ΔE(eV) is the highest transition energy between the upper to a lower ground level (n → m), and T is the excitation temperature in kelvin (K). In this work, the calculated electron number density using this relationship is on the order of ~$10^{14}\;{\mathrm{cm}}^{-3}$. This value of the plasma number density is lower at three orders of magnitude than that determined from Equation (3). Therefore, it can be concluded that plasma is satisfying the LTE state. 

The validity of the LTE condition has also been confirmed by estimating the diffusion length using the Cristoforetti et al. criterion for an inhomogeneous plasma to be in LTE. According to this condition, the characteristic variation length “d” is significantly greater than the diffusion length (10λ <d). The diffusion length was estimated employing the following relation [44,45].

(5) $$\lambda \approx \sqrt{D_{diff}\times \tau_{rel}}=1.4\times 10^{12}\;\left[\frac{{\left(k_{B}T\right)}^{\frac{3}{4}}}{N_{e}}\right].{\left(\frac{\Delta E}{M_{A}\;f_{12}\;\left(\bar{G}\right)}\right)}^{\frac{1}{2}}.\exp\left[\frac{\Delta E}{2k_{B}T}\right].$$

(6) d≈T(x)(dT(x)dx)−1

where Δ*E* and *k_B_T* are determined in electron volt (eV), *N_e_* is the electron density measured in (cm^−3^), *M_A_* denotes the atomic mass of a specie, f_12_ represents the oscillator strength taken from the NIST database [37], $\bar{G}$ is the gaunt factor carefully chosen by Cristoforetti et al. [45], d is the plasma diameter (typically a few mm) and also known as characteristic variation length. In the present work, we have calculated the diffusion length by using the emission line of neutral zinc (Zn I). The calculated diffusion length, $\lambda \;≅\;1.87\times 10^{-3}\;\mathrm{mm}$ is much below the characteristic variational length of the optical plasma (10λ<d), which confirms that the plasma follows the LTE condition. 

### 4.4. Effect of Laser Irradiance on the Plasma Parameters

**Figure 7:**
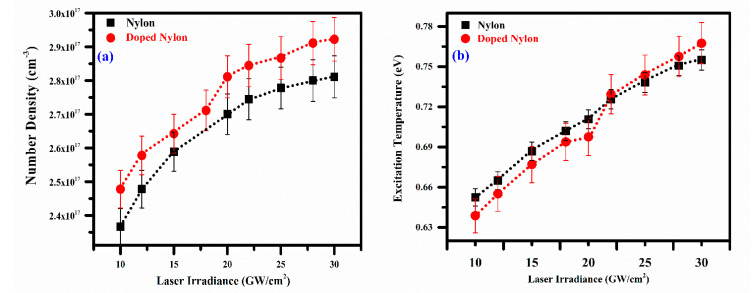
(**a**) Variation in the electron number density and (**b**) Variation in excitation temperature of the pure and doped nylon plasma as a function of laser irradiance.

The effect of electron plasma number density and temperature as a function of laser irradiance was investigated, as shown in Figure 7a,b. The dependency of plasma parameters on the laser irradiance was studied by varying it from 10 to 30 GW/cm^2^. The electron number density for both the samples (pure and doped nylon polymer) is determined through the *FWHA* of the *H_α_* line profile at 656.28 nm and plotted as a function of laser irradiance as shown in Figure 7a. The figure shows that the plasma electron number density increases exponentially from 2.4 × 10^17^ cm^−3^ to 2.8 × 10^17^ cm^−3^ and 2.49 × 10^17^ cm^−3^ to 2.9 × 10^17^ cm^−3^ for pure nylon and co-doped nylon, respectively, at the same laser irradiance from 10–30 GW/cm^2^. Similarly, excitation temperature was determined using the same samples and 532 nm wavelength at various laser irradiances ranging from 10 to 30 GW/cm^2^. Figure 7b demonstrates the variance in electron temperature as a function of laser irradiance from 0.65 eV to 0.76 eV and 0.64 eV to 0.77 eV for pure nylon and co-doped nylon, respectively. The electron temperature is showing almost linear growth concerning laser irradiance due to the sufficient laser absorption onto the target, revealing the inverse bremsstrahlung (IB) effect.

### 4.5. Effect of Laser Irradiance on CN and C_2_ Vibrational Emission

**Figure 8:**
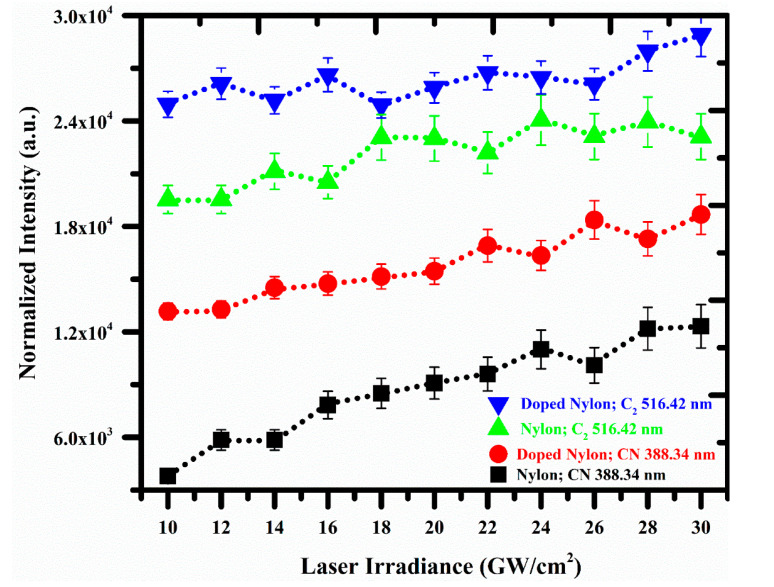
Normalized intensity of CN and C_2_ molecular band at 388.34 nm, and 516.42 nm, respectively, as a function of laser irradiance for pure and doped nylon.

In this study, the intensities of the laser-produced optical plasma of the pure nylon and ZnO/nylon polymer were normalized by dividing the intensity of vibrational lines of CN and C_2_ using the intensity of the neutral carbon (C I) emission spectral line at 247.86 nm under time delay of 2 μs between the laser pulse to the preliminary of the acquisition system, 1 ms integration time and 532 nm laser wavelength. A similar normalization approach has also been investigated in previously reported literature [46,47]. Figure 8 shows the normalized intensity of CN at 388.34 nm and 516.42 nm due to (0,0) vibrational transition as a function of laser irradiance from 10–30 GW/cm^2^ for pure nylon and doped nylon. As shown in the figure, the normalized intensities of the vibrational transitions are growing concerning laser irradiance. The maximum intensity of the CN and C_2_ molecular band for (0,0) vibrational transition is observed at 30 GW/cm^2^ irradiance for both the samples (nylon and doped nylon). At below 10 GW/cm^2^ and above 30 GW/cm^2^, we observed that the signal vanishes for all the modes due to the low absorption of the energy onto the target surface and dissociation of the molecular band structures, respectively. The structural emission at CN: 388.34 nm and C_2_: 516.42 nm are the dominant molecular profiles throughout the whole scale of investigated irradiance from 10–30 GW/cm^2^. In an innovative sense, polymers can be distinguished from the other non-polymer materials corresponding to their vibrational structure. In the nylon sample, the vibrational emission of the CN Violet and C_2_ Swan band due to the (0 → 0) vibrational transition is observed as strongly intense than the lower energy transitions, for instance (1 → 1), (2 → 2), and (3 → 3). Therefore, this factor is significantly very critical when exploring the LIBS spectra of polymers having organic molecular structures. 

## 5. Conclusions

In the present work, molecular emissions, CN (B^2^Σ^+^ to X^2^Σ^+^) violet and the C_2_ (d^3^ П_g_–a^3^ П_u_) swan band system of nylon polymer plasma, at various irradiances from 10–30 GW/cm^2^, were noticed for the vibrational transitions Δν = −1, 0, +1 and Δν = −1, 0, +1, +2, respectively, using a laser-induced breakdown spectroscopy technique. The excitation temperature, plasma electron density, and line intensity as a function of laser irradiance were observed. Depending on the laser pulse irradiance, integration time, excitation temperature, and plasma number density, the characteristics of the CN and C_2_ emission spectra were found to change significantly. These analyses determine that the emission line intensities from CN and C_2_ bands are very sensitive to laser irradiance, integration time and delay time among laser pulse shot and spectral acquisition. At low laser irradiances such as 10 GW/cm^2^, the emission bands due to CN and C_2_ predominate while at higher irradiance the multiple neutral and ionized lines of various elements have been detected together with CN and C_2_ molecular optical emissions. The excitation temperature is noticed to grow with rising laser irradiance and saturates at higher irradiance levels. The saturation of excitation temperature at higher laser irradiance is caused by exhaustion of excited-level population density of CN and C_2_ molecules and by plasma shielding. 

## Figures and Tables

**Table 1:** Spectroscopic atomic parameters of the emission lines for the calculation of excitation temperature [37].

Wavelength *λ* (nm)	Transition Upper to Lower	Upper-Level Energy *E_k_* (eV)	Transition Probability and Statistical Weight *A_k_g_k_* (10^8^ s^−1^)
Ca I			
487.81	4s4f ^1^F_3_ → 3d4s ^1^D_2_	5.25	1.32
527.03	3d4p ^3^P_2_ → 3d4s ^3^D_3_	4.88	2.50
559.85	3d4p ^3^D_1_ → 3d4s ^3^D_1_	4.74	1.29
585.75	4p^2 1^D_2_ → 4s4p ^1^P_1_	5.05	3.30
612.22	4s5s ^3^S_1_ → 4s4p ^3^P_1_	3.91	0.86
649.97	4p ^3^F_2_ → 4s ^3^D_2_	4.43	0.41
671.76	4s5p ^1^P_1_ → 3d4s ^1^D_2_	4.55	0.36
Zn I			
328.23	4d ^3^D_1_ → 4p ^3^P_0_	7.78	2.70
330.29	4d ^3^D_1_ → 4p ^3^P_1_	7.78	6.00
334.50	4d ^3^D_3_ → 4p ^3^P_2_	7.78	11.9
481.05	5s ^3^S_1_ → 4p ^3^P_2_	6.65	0.70

## Data Availability

Not applicable.
